# P-1403. Measuring Impact of COVID-19 Pandemic using Novel EPII Survey on PLWH in New Orleans

**DOI:** 10.1093/ofid/ofae631.1578

**Published:** 2025-01-29

**Authors:** Angela McGaugh, Erik Meyers, Shruti Srinivasan, Mariza Francis, Crystal Zheng, Damion Grasso, Leah Adams, Suzanne Adams

**Affiliations:** Tulane University School of Medicine, New Orleans, Louisiana; LCMC, New Orleans, Louisiana; Tulane University School of Medicine, New Orleans, Louisiana; University of Wisconsin School of Medicine, Madison, Wisconsin; Tulane University, New Orleans, LA; University of Connecticut School of Medicine, West Hartford, Connecticut; Tulane University School of Medicine, New Orleans, Louisiana; Tulane University, New Orleans, LA

## Abstract

**Background:**

Research has shown that the impacts of the COVID-19 pandemic stretch beyond solely physical health. This study uses the newly developed Epidemic-Pandemic Impacts Inventory (EPII), a 92-item tool designed to quantify the impact of COVID-19 pandemic across several personal and social domains. People living with HIV (PLWH) have an increased morbidity and mortality associated with COVID-19 infection and are overall more vulnerable to social change. The current study uses the EPII to compare pandemic-related experiences of PLWH to those without HIV in New Orleans.

**Methods:**

The Tulane University COVID-19 Antibody and Immunity Network (TUCAIN) maintains a longitudinal cohort of PLWH who provide clinical data and biological specimens for studies on the immune response to COVID-19 infection and vaccination. For the current study, participants were asked to complete the EPII assessing their perception of the pandemic. Participants were invited to complete the survey in person or via phone with a study coordinator. Responses from participants ≥18 years old who completed the survey were reviewed. Survey responses were analyzed using SPSS software.

**Results:**

Both the PLWH (N=54) and control group (N=220) were similar in age; however, the PLWH subset was predominantly male and more racially diverse. PLWH reported more severe impacts of pandemic such as food insecurity (χ2=15.9; df=1; p< 0.01) and loss of employment (χ2=26.9; df=1; p< 0.01) and housing (χ2=6.6; df=1; p=0.01). PLWH reported more interruptions in medical care (χ2=12.6; df=1; p< 0.01) and more health problems unrelated to COVID-19 (χ2=7.2; df=1; p< 0.01). The PLWH subset was also less likely overall to report positive impacts of the pandemic (i.e. increased quality time with loved ones) compared to control group.

**Conclusion:**

Results indicate that the HIV cohort reported greater rates of financial and health ramifications of the pandemic relative to control population. This study further reinforces the notion that this population was more susceptible to the socioeconomic turmoil of the COVID-19 pandemic. The results from this study will help to refine EPII as a survey tool for use in future pandemics and more precisely identify domains of social impact that affect the PLWH population the most.

**Disclosures:**

**All Authors**: No reported disclosures

